# Paternal self-efficacy for promoting children’s obesity protective diets and associations with children’s dietary intakes

**DOI:** 10.1186/s12966-019-0814-5

**Published:** 2019-06-28

**Authors:** Adam D. Walsh, Kylie D. Hesketh, Jill A. Hnatiuk, Karen J. Campbell

**Affiliations:** Institute for Physical Activity and Nutrition (IPAN), School of Exercise and Nutrition Sciences, Deakin University, Geelong, Victoria Australia

**Keywords:** Fathers, Diet, Early childhood, Self-efficacy, Parenting

## Abstract

**Objective:**

Fathers’ parenting behaviours contribute to the development of children’s dietary behaviours and subsequent weight outcomes, yet the majority of research focusses on maternal influences. Understanding fathers’ perceptions of their effectiveness to influence children’s dietary behaviours will allow the development of whole-of-family interventions promoting obesity protective behaviours. This unique study is the first to investigate 1) tracking of paternal self-efficacy for promoting obesity protective dietary intakes in young children; 2) demographic characteristics of fathers and their self-efficacy category; and 3) associations between paternal self-efficacy and young children’s dietary intakes.

**Methods:**

Paternal self-efficacy for promoting children’s obesity protective dietary intakes was assessed longitudinally from fathers (*n* = 195) in the Extended Infant Feeding Activity and Nutrition Trial Program at child age 4 and 36 months. Multinomial logistic regression examined self-efficacy tracking categories (persistently high; persistently low; increasing; decreasing) by paternal age, education and BMI. Linear regression examined associations between paternal self-efficacy tracking categories and child dietary intakes at 36 months.

**Results:**

Paternal self-efficacy for promoting children’s obesity protective dietary intakes reduced over time. Fathers with trade/certificate or university qualifications had lower odds of having persistently low/decreasing self-efficacy (97 and 87% lower respectively) compared to high-school educated fathers. Positive associations (β (95% CI)) were observed between paternal self-efficacy category and children’s dietary intakes at 36 months: increasing self-efficacy and fruit (β89.8 (6.8; 172.7)), and vegetables (β39.2 (12.2; 66.2)); persistently high self-efficacy and water (β69.1 (2.9; 135.1)); decreasing self-efficacy and non-core drinks ((β30.1 (10.1; 50.1)). Persistently high self-efficacy was negatively associated with non-core drinks (β-20.2 (− 34.8; − 5.5)), with negative associations observed between decreasing self-efficacy and children’s intakes of fruit (β − 49.9 (− 87.5; − 12.3)), vegetables (β-19.9 (− 31.7; − 8.2)) and water (β-92.4 (− 172.6; − 12.3)).

**Conclusions:**

Higher and/or sustained paternal self-efficacy is associated with fathers’ education and is important in promoting children’s obesity protective dietary intakes. Associations between paternal self-efficacy and children’s dietary intakes are present at a young age. This investigation was unique in its focus on paternal self-efficacy for promoting children’s obesity protective dietary intakes and associations with children’s dietary intakes. Future family interventions should consider how to maintain and/or improve paternal self-efficacy to promote obesity protective intakes from early childhood.

## Background

Children’s dietary intakes have been shown to be low in core foods such as fruit and vegetables and high in nutrient-poor, energy-dense foods [1–3]. Evidence suggests this is the case even in pre-school aged children [3–7]. The subsequent impact of these dietary intakes on overweight and obesity in childhood and adolescence have adverse health consequences in adult life, with these health consequences significantly increasing risk of chronic disease and early mortality [8]. There has been little work involving fathers in the prevention or treatment of childhood obesity [9]. Previous research has found that a variety of paternal factors, including their own dietary intake [10, 11], general parenting style and parenting behaviours [12] are associated with children’s dietary intakes and/or weight outcomes. However, little research exists on how fathers perceive their ability to facilitate or limit children’s dietary intakes. This is likely to provide valuable insights for the development of effective family-based interventions to promote children’s energy balance behaviours.

First-time parents may have little to no experience in infant feeding, although they are likely to have thoughts or knowledge about what constitutes healthy behaviours [13]. First-time parents regularly seek advice during their child’s first year of life [14] which reflects parental need for support and information during this period of rapid child growth and transition. Parents often experience concern regarding children’s eating and growth, and may seek guidance from a variety of sources [15]. Accordingly, first-time parents may be receptive to knowledge and skill development relating to parenting, including the promotion of obesity protective behaviours. Self-efficacy describes a person’s confidence to engage in a particular behaviour in a particular situation [16]. Social Cognitive Theory suggests that parenting behaviours around children’s dietary intakes are a result of the interaction between parental perception of their ability to perform the new behaviour (self-efficacy), parental beliefs, including the value of the new behaviour (outcome expectations), and the social and physical environment in which parenting occurs [17, 18]. There is scant evidence regarding paternal self-efficacy and child dietary intakes, with research involving fathers restricted to investigating parenting self-efficacy more generally (i.e. competence in the parental role) [19, 20]. In contrast, maternal self-efficacy has been well explored in the parenting literature [21–24], including in the context of children’s energy balance behaviours [25–28].

Investigations of maternal self-efficacy suggest that higher maternal self-efficacy may facilitate obesity protective energy balance behaviours in young children [26, 28] but that maternal self-efficacy may also decline as children become older [26, 28]. For example, in their cross-sectional study of maternal self-efficacy in mothers of one-year old (*n* = 60) and five-year old (*n* = 80) children, Campbell and colleagues [28] observed that it was mothers of one-year old children who reported greater confidence to limit undesirable energy balance behaviours. Higher maternal self-efficacy was associated with more favourable child intakes; higher child consumption of water, fruits, and vegetables at 5 years of age, and higher vegetable consumption and lower consumption of cordial and cake at 1 year of age. In the context of child sedentary behaviours, Hnatiuk and colleagues [26], investigated tracking of maternal self-efficacy for limiting children’s television viewing at child age 4 and 19 months in a cohort of 404 Australian families. They observed that tracking of maternal self-efficacy for limiting television viewing was low, but those mothers who had persistently high or increasing self-efficacy had children who watched 35 min less television at 19 months of age when compared to children of mothers with persistently low self-efficacy [26].

The relationship between paternal self-efficacy and young children’s dietary intakes, as well as tracking of paternal self-efficacy for facilitating obesity protective dietary intakes in young children, are likely to have important implications for both the design and the timing of delivery of family-based obesity-prevention interventions. Given their status as first-time fathers, measuring paternal self-efficacy for facilitating obesity protective dietary intakes in young children, prior to actual child-feeding experiences, and again at a later time-point once child-feeding is established, may provide valuable insights into what changes occur in paternal self-efficacy, and how best programs may be developed to support fathers to maintain (or increase) their self-efficacy. Accordingly, the aims of this study were to examine: 1) tracking of paternal self-efficacy for promoting obesity protective dietary intakes in children aged four to 36 months; 2) demographic characteristics of fathers who maintained, increased or decreased their self-efficacy for promoting obesity protective dietary intakes in young children over the first 36 months of their children’s lives; and 3) associations between paternal self-efficacy for promoting obesity protective dietary intakes in young children and young children’s dietary intakes.

## Methods

### Participant recruitment

This study performed secondary analysis of data collected from fathers (*n* = 195) participating in the extended Infant Feeding, Activity and Nutrition Trial (InFANT Extend) Program when children were approximately four and 36 months of age. The InFANT Extend Program was a cluster randomised controlled trial of a parent-focused child obesity prevention intervention, the details of which have been reported elsewhere [29]. Briefly, the trial aimed to test the effectiveness of a group-based program (six sessions) delivered to first time mothers when infants were approximately 3–18 months of age. Quarterly newsletters were provided to participants when children were 18–36 months of age. Control participants received usual care and general health newsletters. Recruitment of participants occurred from first-time parent groups in seven local government areas (LGA) within a 75 km radius of the research centre (Geelong, Victoria, Australia).

Eligibility to participate included being a first-time parent and English literacy. Both the main carer and partner were invited to participate in the program however, with only main carers (mothers) participating, fathers were not directly exposed to the program. Infants with chronic health problems likely to influence height, weight, levels of physical activity or eating behaviours were permitted to participate in the program but were excluded from analyses. The InFANT Extend Program was approved by the Deakin University Human Research Ethics Committee (EC-175-2007; Part 2–2007–175) and the Department of Education and Early Childhood Development (Victoria, Australia) (2011_001000). Informed written consent was obtained from all participants.

### Measures

Fathers completed paper questionnaires providing demographic and socio-economic variables including date of birth to calculate age; country of birth; main language spoken at home; employment status and education level. Self-reported weight and height were collected with body mass index (BMI) calculated as weight (kg)/height (m^2^). Paternal BMI was split into three groups (healthy weight, overweight and obese) [30]. A dichotomous paternal age variable was created, split at the median age of the fathers in the sample (33.4 years) [10]. Paternal education was collapsed into three groups (high school or lower; trade or certificate qualification; university qualification) [31].

Paternal self-efficacy for promoting young children’s obesity protective dietary intakes was assessed using four items from a previously developed scale as, at the time of data collection, no measures to assess self-efficacy in these domains were published [28]. Fathers were asked about their confidence regarding their children’s fruit, vegetable, water and non-core drink intake over the next year with responses using a four point Likert scale from 1; ‘not at all confident’ to 4; ‘extremely confident’ (e.g.: “How confident are you that you will be able to get your child to drink plain water over the next year?”). A self-efficacy score was then generated by averaging the scores of the four items. This score had good internal reliability in this sample (α = 0.88 at child age 4 months (T1) and α = 0.75 at child age 36 months (T2)).

The paternal self-efficacy score was split into quartiles at T1 and T2 separately. A categorical variable was created, using a similar approach to other tracking studies [26, 32, 33], to assess the direction of any self-efficacy change. This variable contained four categories: persistently high self-efficacy (high or very high quartiles at T1 and T2); persistently low self-efficacy (low or very low quartiles at T1 and T2); increasing self-efficacy (from low or very low quartiles at T1 to high or very high quartiles at T2); decreasing self-efficacy (from high or very high quartiles at T1 to low or very low quartiles at T2).

Children’s dietary intakes were assessed when they were 36 months of age using a 66 item food frequency questionnaire (FFQ) completed by the main carer (mother), with data analysed using the 2007 Australian Food and Nutrient Database (AUSNUT) Database [34]. Dietary outcome variables included in analyses were those corresponding to the parent survey dietary self-efficacy questions (fruit, vegetables, water, non-core drinks). The average daily intake (in grams) of vegetables (12 items), fruit (12 items; excluding juice), as well as millilitres of water (2 items) and non-core drinks (10 items) (i.e. fruit juice, soft drinks) were calculated [29].

### Statistical analyses

As there were no differences in paternal self-efficacy for promoting young children’s obesity protective dietary intakes between intervention and control groups, data were pooled. Tracking of paternal self-efficacy was assessed using multinomial logistic regression with persistently high self-efficacy at T1 and T2 used as the reference category. The odds of being in different self-efficacy categories based on paternal demographic predictors were analysed using multinomial logistic regression (reference category: persistently high self-efficacy). Associations between fathers’ self-efficacy tracking category and children’s fruit, vegetable, water and non-core drink intakes were assessed using linear regression analyses. All analyses were adjusted for intervention status and the cluster-based sampling design (first-time parent groups). The significance level was set at 5%. Analyses were conducted using Stata software (release 15; StataCorp LP, College Station, TX, USA).

## Results

The sample available at baseline (child age approximately 4 months) was 433 fathers. Follow-up data were missing for 238 fathers, thus the final sample with complete data at child age approximately 36 months consisted of 195 fathers (45%). Mean paternal age was 34.7 (SD 5.5) years. The majority of the sample were born in Australia (79.5%). Similar proportions of fathers were university (42.4%) or trade/certificate (39.8%) qualified with the remainder (17.8%) reporting either some high school or high school completion as their highest level of education. The majority of fathers in this study were overweight (50.2%), whilst 33.9 and 15.9% were in the healthy weight and obese categories respectively. There were no significant differences in baseline characteristics (age, education, weight, self-efficacy category) between those lost to follow-up and those retained at 36 months.

**Fig. 1:**
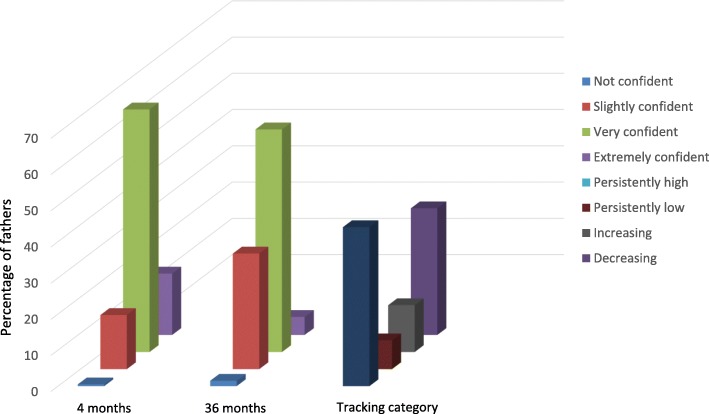
Paternal self-efficacy categories for promoting children’s obesity protective dietary intakes between 4 and 36 months

At baseline, approximately 0.5% of fathers were categorised as not confident, 15% as slightly confident, 67% as very confident and 17% extremely confident. Figure 1 presents the percentage of fathers within each category of paternal self-efficacy at each time point and prevalence of each self-efficacy tracking category. The majority of fathers were categorised in either the persistently high self-efficacy category (44%) or decreasing self-efficacy category (35%) between child age four to 36 months. There was a significant decrease in the mean average paternal self-efficacy scores between child age four and 36 months (3.2 (SD 0.4) vs 2.9 (SD 0.5); *p* = 0.02).

Relative to fathers who had a high school education only, those fathers who were trade/certificate qualified had 97, 84 and 94% lower odds of having persistently low, decreasing or increasing self-efficacy, respectively, for promoting young children’s healthy dietary intakes compared to fathers with persistently high self-efficacy (Table 1). Additionally, relative to fathers who had a high school education only, those fathers who were university qualified had 87, 73 and 76% lower odds of having persistently low, decreasing or increasing self-efficacy respectively, compared to fathers with persistently high self-efficacy. No difference in odds were observed for paternal age or BMI.

**Table 1:** Fathers’ self-efficacy category to promote healthy eating according to paternal predictors^a^

Paternal characteristics	Persistently low self-efficacy		Decreasing self-efficacy		Increasing self-efficacy	
	OR	95% CI	OR	95% CI	OR	95% CI
Age						
≤33.4 years	1.0 (ref)		1.0 (ref)		1.0 (ref)	
≥33.5 years	0.34	(0.10–1.15)	1.11	(0.60–2.10)	1.00	(0.41–2.47)
Education						
High school or lower	1.0 (ref)		1.0 (ref)		1.0 (ref)	
Trade or certificate	**0.03**	**(0.004–0.19)**	**0.16**	**(0.05–0.50)**	**0.06**	**(0.01–0.26)**
University	**0.13**	**(0.03–0.53)**	**0.27**	**(0.09–0.84)**	**0.24**	**(0.06–0.95)**
BMI category						
Healthy weight	1.0 (ref)		1.0 (ref)		1.0 (ref)	
Over weight	2.12	(0.53–8.37)	0.82	(0.40–1.67)	0.65	(0.25–1.71)
Obese	1.60	(0.24–10.81)	1.58	(0.62–4.04)	0.46	(0.08–2.44)

Multinomial logistic regression, adjusted for intervention group and clustering by first-time parent group (reference group persistently high self-efficacy)

^a^Bold results are significant at *p* < 0.005

Associations between paternal self-efficacy tracking category and children’s dietary intakes at 36 months of age are reported in Table 2. Persistently high self-efficacy was associated with higher water intake and lower non-core drink intake. Increasing self-efficacy was associated with higher fruit and vegetable intake and lower non-core drink intake. Decreasing self-efficacy was associated with lower consumption of fruit, vegetables and water, and higher consumption of non-core drinks. Fathers’ self-efficacy accounted for 36% of the explained variance (Table 2).

**Table 2:** Associations between fathers’ self-efficacy tracking category and children’s dietary intakes (β (95% CI)^a^

Food group	Persistently high self-efficacy	Increasing self-efficacy	Decreasing self-efficacy	Persistently low self-efficacy
Fruit	8.9 (− 32.2; 50.03)	**89.8 (6.8; 172.7)**	**-49.9 (− 87.5; − 12.3)**	−9.5 (− 127.8; 108.9)
Vegetables	4.3 (− 9.7; 18.3)	**39.2 (12.2; 66.2)**	**−19.9 (−31.7; − 8.2)**	−11.3 (− 35.1; 12.4)
Water	**69.1 (2.9; 135.1)**	45.8 (− 43.2; 134.7)	**−92.4 (− 172.6; − 12.3)**	−15.1 (− 108.9; 78.7)
Non-core drinks	**−20.2 (− 34.8; − 5.5)**	−15.5 (− 32.9; 1.8)	**30.1 (10.1; 50.1)**	−2.2 (− 22.8; 18.4)
% variance explained	6.0%	12.8%	17.2%	< 1%

All variables adjusted for intervention status and clustering

Intake was measured in grams/millilitres per day

^a^Bold results are significant at *p* < 0.005

## Discussion

This study was the first to investigate paternal self-efficacy in relations to young children’s dietary intakes. It provides important insights into how paternal self-efficacy changes over time, as well as the impact of paternal self-efficacy on young children’s obesity protective dietary intakes. Accordingly, this study highlights the importance of considering paternal (alongside maternal) self-efficacy in the context of young children’s dietary intakes.

More fathers reported reduced rather than increased self-efficacy over the first 3 years of life which is consistent with work investigating maternal self-efficacy and child energy balance behaviours [26, 28]. Hnatiuk and colleagues [26] observed low tracking of maternal self-efficacy for limiting children’s television viewing between child age 4–19 months in their sample of over 400 Australian mothers. Additionally, Campbell and colleagues [28], in their cross-sectional study of mothers of one and five-year-old children, observed lower maternal self-efficacy for limiting non-core foods and screen-time in mothers of five-year-old children compared with mothers of infants. Whilst the current study, along with the previous two studies, used the same tool to measure parental self-efficacy, this accumulating evidence, across different samples, provides consistent evidence regarding decreasing parental self-efficacy during early childhood. These findings suggest family-based interventions are needed to maintain parental self-efficacy given higher parental self-efficacy is associated with more favourable child health outcomes and behaviours [26, 35, 36].

Our observation of an overall decrease in fathers’ self-efficacy may have been influenced by the first-time father status of the sample. Fathers with more than one child may display a different self-efficacy profile. [37] It may be that prior child rearing experiences influence fathers’ self-efficacy with subsequent children (possibly higher or lower self-efficacy depending on previous experience) [28]. The timing of the decrease in paternal self-efficacy cannot be determined in the current study given the 32 month period between measures. It may be that self-efficacy decreases continuously across a child’s life; soon after the introduction of solid foods; or when toddler eating behaviours such as food refusal or fussy eating have emerged. Nevertheless, the implementation of targeted approaches to maintain or increase paternal self-efficacy likely need to commence as early as possible, possibly prior to the introduction of solid foods, so as to maximise any impact of such approaches. Indeed, other work in parenting self-efficacy has demonstrated successful outcomes in improving first-time parent self-efficacy, [20] and young child dietary intakes, [38] suggesting it is feasible to do so. Given the known associations between paternal and young child dietary intakes [11], the development of whole-of-family health promotion programs that aim to maintain or increase paternal self-efficacy during early childhood are likely to be important.

The influence of parental education status has been previously investigated in the context of parental feeding practices [39] and associations between parents’ and children’s dietary intakes [10, 40]. Paternal education status has not previously been investigated in the context of fathers’ self-efficacy for the promotion of young children’s obesity protective dietary intakes. Our observation that fathers with a trade/certificate or university qualification were less likely to have persistently low self-efficacy or decreasing self-efficacy for promoting young children’s healthy dietary intakes when compared to those whose highest educational attainment was secondary school is consistent with cross-sectional work by Ekim [41] involving 3–6 year old Turkish children and their mothers. That study observed increasing maternal self-efficacy scores with increasing education status. Whilst our study observed decreasing paternal self-efficacy over time among fathers in all education groups, fathers who were trade/certificate or university qualified were less likely to report decreasing self-efficacy. This finding may suggest that interventions that aim to maintain or increase paternal self-efficacy may be even more important for fathers who have no formal qualification.

Our observation of associations between persistently high, as well as increasing, paternal self-efficacy and obesity protective child dietary intakes is consistent with findings from two previous cross sectional studies from Parekh and colleagues [36] and Ice and colleagues [35]. The Parekh study observed positive associations between higher parental self-efficacy and child fruit intake and inverse associations between higher parental self-efficacy and child intakes of non-core snacks and sugar sweetened beverages [36], while the Ice study observed correlations between higher parental self-efficacy and child intake of fruit and vegetables [35].

The current study adds to the evidence base by focusing on fathers’ self-efficacy specifically. The previous study samples have included only 22% [36] and 8% [35] of fathers, respectively, and analysed pooled parental data. Our results, consistent with those of maternal-dominated studies, indicate that the self-efficacy of fathers is also an important influence on children’s obesity protective behaviours.

Our investigation was novel in its focus on paternal self-efficacy for promoting young children’s obesity protective dietary intakes and associations with young children’s dietary intakes. To our knowledge, this is the first study to assess these concepts in fathers of young children. Study strengths included the longitudinal design, diverse socioeconomic sample despite loss to follow-up, and the reliability of the self-efficacy measure as shown by test-retest. There were some limitations to the study that should be noted. Dietary data were proxy reported for children (by mothers) and are therefore susceptible to social desirability bias. However, as the focus was the association between paternal and child data, associations should still be apparent even in the presence of a bias toward socially desirable reporting. We acknowledge that the percentage of variance is low, indicating other factors not measured in this study influence children’s intake and care should be taken when interpreting wide confidence intervals observed for the dietary intake data. Whilst this study aimed to elucidate predictors of paternal self-efficacy based on previous literature, it is possible that other self-efficacy predictors not measured within the current study (e.g. parenting stress, marital satisfaction) may also impact on paternal self-efficacy. A further limitation was the relatively small sample size and participant loss to follow-up, although participant attrition does appear random. Finally, the study results might not be generalizable to fathers with more than one child given the focus on first-time fathers.

## Conclusions

Our results show that paternal self-efficacy for promoting young children’s obesity protective dietary intakes changes over a two and a half year period in a mostly downward direction. Additionally, we observed important associations between higher levels of paternal self-efficacy for promoting young children’s obesity protective dietary intakes and more favourable child dietary intakes at 36 months of age. These findings highlight the potentially important role fathers’ self-efficacy may play in determining child dietary intakes. Specifically, findings from the current study suggest that support for fathers is warranted to both increase, and prevent decreasing, paternal self-efficacy in the context of promoting young children’s healthy dietary intakes. Future research, as well as including examination of both parents’ influences on children’s dietary intakes, could investigate the strategies employed by fathers who attain and maintain high self-efficacy for promoting young children’s healthy dietary behaviours is warranted in an effort to better understand how these strategies assist fathers in maintaining their self-efficacy. Additionally, investigation of when paternal self-efficacy begins to decrease will allow the delivery of time sensitive interventions that increase paternal self-efficacy and enable the development of obesity protective family environments.

## Data Availability

The datasets used and/or analysed during the current study are available from the corresponding author on reasonable request.
